# The incidence, monitoring coverage and clinical characteristics of hydroxychloroquine retinopathy in the United Kingdom

**DOI:** 10.1038/s41433-024-03168-0

**Published:** 2024-07-31

**Authors:** Imran H. Yusuf, Ruofan C. Han, Susan M. Downes, Srilakshmi M. Sharma

**Affiliations:** 1 Nuffield Laboratory of Ophthalmology, Department of Clinical Neurosciences, Oxford University, West Wing, John Radcliffe Hospital, Headley Way, Oxford, OX3 9DU UK; 2 Oxford Eye Hospital, John Radcliffe Hospital, Oxford University Hospitals NHS Foundation Trust, Headley Way, Oxford, OX3 9DU UK

**Keywords:** Retinal diseases, Vision disorders

## Abstract

**Background:**

Retinal monitoring is recommended for hydroxychloroquine users to detect pre-symptomatic retinopathy and preserve visual function. However, the incidence of hydroxychloroquine retinopathy and monitoring coverage in the U.K. are incompletely characterised. Moreover, the visual benefits of monitoring for retinopathy – recommended for over 70,000 long-term hydroxychloroquine users in the U.K. - remain unproven.

**Methods:**

A national, prospective observational study was undertaken with the British Ophthalmological Surveillance Unit (BOSU). Newly diagnosed cases of hydroxychloroquine retinopathy in the U.K. were reported and data captured using a standardised questionnaire over 3.5 years (July 2018–Dec 2021). The frequency of retinopathy and coverage of monitoring amongst long-term users was estimated. Visual function was compared between asymptomatic individuals detected on monitoring and those presenting with visual symptoms. The clinical characteristics, dosing and management of reported cases were captured.

**Results:**

The annualised number of incident cases of hydroxychloroquine retinopathy was 29–57, with an annualised frequency of 0.04–0.08% amongst long-term users (~1 in 1247–2625). The coverage of monitoring was approximately 2.6–5.5%. Visual acuity (0.1 vs. 0.22 logMAR; *p* = 0.007) and visual field mean deviation (−3.73 dB vs. −8.69 dB; *p* = 0.017) were better preserved in asymptomatic individuals compared to those presenting with visual symptoms.

**Conclusion:**

These data support the efficacy of monitoring in the preservation of visual function in patients with hydroxychloroquine retinopathy at diagnosis. The overall population coverage of monitoring was low, consistent with the high proportion of symptomatic patients at diagnosis. This study presents a method for evaluating the yield of monitoring for hydroxychloroquine retinopathy in the U.K.

## Introduction

Hydroxychloroquine retinopathy is an increasingly recognised cause of irreversible, bilateral visual loss in users of hydroxychloroquine and other 4-aminoquinolines [1, 2]. Although hydroxychloroquine was originally developed as an anti-malarial agent, its broad biological activities have subsequently led to its wide repurposing for an expanding group of clinical indications [2]. A survival benefit of hydroxychloroquine has been identified in patients with systemic lupus erythematosus [3], encouraging its use in all patients in this group. The efficacy, low cost and favourable systemic safety profile of hydroxychloroquine has led to a significant increase in the number of long-term users in developed nations over the past 20 years [4].

Increasing access to modern retinal imaging techniques over this period, principally optical coherence tomography (OCT), has enabled the detection of pre-symptomatic retinopathy [5, 6]. In the pre-OCT era, the frequency of detectable retinopathy in long-term hydroxychloroquine users (>5 years duration) was approximately 0.5%, when the disease was severe enough to be evident on clinical examination [7, 8]. At this stage, patients are typically symptomatic, and disease progression is highly likely despite drug cessation [6, 9–11]. In the OCT era, the frequency of retinopathy is approximately 5–10% amongst long-term hydroxychloroquine users in retrospective studies that define disease as a single abnormal test result consistent with retinopathy [12–14]. However, real world audit data from the United Kingdom suggest a frequency of “definite retinopathy” of approximately 1.5% in long-term users from aggregated data when disease was defined as two abnormal test results consistent with toxicity [12, 15, 16].

The American Academy of Ophthalmology (AAO; 2011, 2016) [17, 18] and the Royal College of Ophthalmologists (RCOphth; 2018 & 2020) [19, 20] recommend annual screening or monitoring (the preferred term in the U.K.) in long-term hydroxychloroquine users. The recommendations aim to detect the earliest pre-symptomatic manifestations of retinal toxicity in order to limit the degree of functional impairment at diagnosis and to reduce the risk of disease progression following dose reduction or drug cessation. However, it has not yet been shown that visual function is better preserved at the point of diagnosis in patients who received retinal monitoring procedures versus those who did not.

The primary aim of this study was to identify the incidence of hydroxychloroquine retinopathy in the United Kingdom (U.K.) – both in patients at risk, and at the population level - and to determine the approximate coverage of monitoring services in the U.K. using published data on the number of long-term hydroxychloroquine users. The secondary aim of the study was to compare visual function between asymptomatic patients detected on retinal monitoring procedures and symptomatic patients with hydroxychloroquine retinopathy. The study was initiated shortly after new monitoring recommendations were published in the U.K. (2018), enabling this comparison. The final aim was to describe the demographics, treatment indications, risk characteristics, clinical features, diagnostic modalities, and management of hydroxychloroquine retinopathy in the United Kingdom.

## Materials and methods

A prospective epidemiological study of hydroxychloroquine retinopathy diagnosed in the hospital eye service was conducted through the British Ophthalmological Surveillance Unit (BOSU) of the RCOphth, London. The study was approved by the UK national research ethics committee in the United Kingdom (ref: 17/SC/0574) and adhered to the tenets of the Declaration of Helsinki.

Monthly reporting cards were sent to all permanently employed Consultant and Associate Specialist Ophthalmologists (~1420) in the United Kingdom, who were asked to report newly diagnosed cases of hydroxychloroquine retinopathy. The case definition was stated as: “any case of bilateral retinal dysfunction in a patient taking hydroxychloroquine in whom hydroxychloroquine retinopathy is thought to be the principal cause.” Reporting cards were returned as either reporting an encountered case, or none. Case ascertainment was undertaken over a total period of 3.5 years (July 2018 to December 2021). Due to the impact of COVID-19 on hospital outpatient services, incidence calculations were derived from cases diagnosed during a continuous 20-month period preceding the pandemic (July 2018 to February 2020) (Supplementary Fig. 1). Clinical data from all reported cases over the 3.5-year study period were otherwise included.

The study investigators sent the reporting clinicians a questionnaire (Supplementary Table 1) to identify demographic details, date of diagnosis of hydroxychloroquine retinopathy, drug indication, daily dose and duration of hydroxychloroquine use, other risk factors for retinopathy, distribution of retinopathy (i.e. parafoveal, pericentral or mixed/generalised), presence or absence of visual symptoms, visual function (best corrected visual acuity (BCVA) and mean deviation on automated central static perimetry), ocular co-morbidities, investigations used to confirm the diagnosis and initial management. Cases that did not meet the case definition were excluded. Body weight was not included since it is not routinely measured in the eye clinic. Reminders were sent to reporting Ophthalmologists in the event of non-returned questionnaires. Follow-up questionnaires were sent out to all respondents 12 months after the initial date of diagnosis, or if the patient had been diagnosed prior to the reporting window, 12 months after receipt of the initial questionnaire. They sought to determine the final management decision, clinical status, visual function and eligibility for sight impairment or severe sight impairment at follow-up.

Statistical analysis was performed using GraphPad Prism version 9.2.0 (GraphPad Software, California, USA).

## Results

### Case ascertainment and filtering

Over a 3.5-year study period, 123 cases of hydroxychloroquine toxicity were reported (Supplementary Fig. 1). Personal communication was received regarding two unreturned questionnaires where the diagnosis had changed. Two further questionnaires did not meet the case inclusion criteria, and one report was duplicated. 118 study questionnaires were sent to reporting consultants, of which 80 were returned (67.8% response rate). Data were scrutinised against the case definition; exclusions included two cases which did not match the case definition, two due to duplication, and three due to quinine toxicity. 73 confirmed cases of hydroxychloroquine retinopathy were included for subsequent analysis.

### Incidence of hydroxychloroquine retinopathy

49 incident cases of hydroxychloroquine retinopathy were identified during the period of July 2018 to February 2020 (Table 1 & Supplementary Figs. 1 and 2), a raw, unadjusted incidence of 29.4 newly diagnosed cases per year in the U.K (Table 1). Adjusting for the under-ascertainment (76% BOSU report card return rate) [21] and under-reporting in this study (67.8% response rate), the likely range of cases of hydroxychloroquine retinopathy presenting to the hospital eye service annually during this period was 29 to 57 cases (Table 1). Based on published estimates of long-term hydroxychloroquine users in the UK during this period (71,144–77,170) [22], these data suggest a maximum annualised frequency of incident retinopathy amongst long-term users of 1 in 1247 (0.08%) during the initial 20-month interval (predicted range: 1 in 1247–2625 (0.04–0.08%); Table 1). The incidence of hydroxychloroquine retinopathy in the general adult population of the United Kingdom was approximately 0.56–1.09 cases per million, per year, based on a UK adult population estimate of 52.4 million in 2018. The incidence for the entire U.K. population was approximately 0.44–0.86 cases per million, per year, based on a total population estimate of 66.5 million in 2018 (Office for National Statistics, 2022) (Table 1).

**Table 1:** Estimates of incidence and prevalence of hydroxychloroquine retinopathy and monitoring coverage in the UK.

Frequency of hydroxychloroquine retinopathy	
Cases reported (July 2018–Feb 2020)	49
Annualised number of reported cases (raw)	29.4
Annualised number of reported cases (adjusted for under-reporting & under-ascertainment)	57.1

Frequency of hydroxychloroquine retinopathy amongst long-term users in the U.K.		
Long-term user estimates [22]	Annualised frequency of detected retinopathy amongst long-term hydroxychloroquine users in the U.K.	
	Min (raw)	Max (adj.)
71,144	0.04%	0.08%
(Clinical Practice Research Datalink)	1 in 2420	1 in 1247
77,170	0.04%	0.07%
(NHS Digital)	1 in 2625	1 in 1353

Population incidence of hydroxychloroquine retinopathy in the U.K.			
	Annualised number of reported cases	Population incidence (cases/million/year)	Population frequency
Total population	29.4 (min)	0.44	1 in 2,260,544
66,460,000	57.1 (max)	0.86	1 in 1,165,965
Adult population	29.4 (min)	0.56	1 in 1,782,427
52,403,344	57.1 (max)	1.09	1 in 919,357

Real world estimates of frequency of definite retinopathy in long-term users in the U.K.			
	Affected	Examined	Frequency of retinopathy
Marshall et al. [12]	14	869	1.6%
Alieldin et al. [15]	10	566	1.77%
Gobbett et al. [16]	2	333	1%
*Aggregated data*	*26*	*1,768*	*1.47%*

Estimated number of affected individuals and coverage of hydroxychloroquine retinopathy monitoring in the U.K. (July 2018–Feb 2020)			
Long-term users	Estimated no. of affected individuals at 1.47%	Coverage of monitoring: Min (raw)	Coverage of monitoring: Max (adj)
71,144	1,046	2.81%	5.45%
77,170	1,135	2.59%	5.03%
Estimated annualised number of individuals screened (July 2018–Feb 2020)			1,999 - 3,880

### Estimation of hydroxychloroquine monitoring coverage

Aggregated real world audit data of monitoring outcomes in patients at risk of hydroxychloroquine retinopathy (i.e. greater than 5 years of exposure) from three published UK studies [12, 15, 16] identified definite retinopathy (RCOphth definition, as used in this study) [19, 20] in 26 of 1768 monitored individuals (an average frequency of 1.47%; Table 1). At this frequency, published estimates of 71,144 to 77,170 long-term hydroxychloroquine users (>5 years) in the U.K. suggest approximately 1046 to 1135 individuals in the population with hydroxychloroquine retinopathy (either detected or undetected).

In the initial study period, the annualised rate of diagnosis of retinopathy as a proportion of the total number of expected patients with hydroxychloroquine retinopathy in the adult population of the UK was 2.59% to 5.45% - this range represents the estimated annualised coverage of monitoring services in the UK during the initial study period (July 2018 to February 2020) amongst at risk individuals (>5 years exposure), assuming the rate of detection of retinopathy is consistent with published U.K. audit data. Using this method, it is likely that approximately 1999–3880 at-risk individuals underwent retinal monitoring annually during this period (Table 1).

### Demographic characteristics of reported cases

**Fig. 1:**
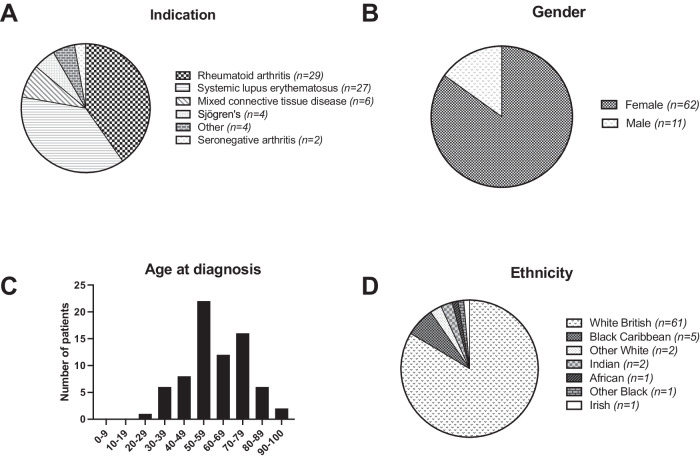
Demographic characteristics of patients with hydroxychloroquine retinopathy. **A** Indication for hydroxychloroquine use. “Other” causes include non-specific interstitial pneumonitis (*n* = 1), primary biliary cholangitis (*n* = 1), CREST syndrome (*n* = 1), and malaria (*n* = 1). **B** Gender of reported cases. **C** Age distribution of hydroxychloroquine retinopathy at diagnosis. **D** Ethnicity of reported cases.

Demographic information is presented in Fig. 1, with clinical parameters and dosing characteristics of reported patients summarised in Table 2. Datasets were obtained for 73 patients. In the cohort, there were 62 females and 11 males. The average age at diagnosis was 61.1 years, with a range of 28–94 years (standard deviation of 15.2 years). Treatment indications are presented in Fig. 1.

**Table 2:** Summary of patient characteristics.

**Characteristics of cohort**				
Mean age at diagnosis (range, *SD*)	61 years (28–94, *15.2*)			
Median visual acuity at diagnosis logMAR (range, *IQR*)	OD	OS		
	0.10 (−0.1 to 1, *0 to 0.27*)	0.10 (−0.20 to 1.8, *0 to 0.29*)		*P* = 0.29
Median 10-2 Humphrey visual field MD (dB) (range, *IQR*)	−4.8 dB (−29.96 dB to −0.05 dB, −*10.39* *dB to −2.42* *dB*)	−3.92 dB (−29.30 dB to 1.30 dB, *−8.95* *dB to −2.22* *dB*)		*P* = 0.71
Disease distribution	Parafoveal	Pericentral	Mixed	
	50	6	7	
Median daily dose, mg (range, *IQR*)	350 mg (66–400 mg, *200–400* *mg*)			
Median duration of drug use (range, *IQR*)	12 years, 1 month (4 months to 50 years, *9 years 1.5 months to 20 years*)			
ELM disruption on OCT imaging	63%			
**Comparison of symptomatic and asymptomatic patients**				
	**Symptomatic**	**Asymptomatic**		
Number	27	43		
Mean age, years (range, *SD*)	60.5 years (14.7 years, *30–82 years*)	61.8 years (15.4 years, *28–94 years*)		*P* = 0.73
Median duration of drug use, years (range, *IQR*)	12 years (3 years to 25 years, *8 years 0.75 months to 20 years*)	13 years (4 months to 50 years, *9 years 9 months to 20 years*)		*P* = 0.33
Median daily dose, mg (range, IQR)	400 mg (200–400 mg, *200–400 mg*)	250 mg (66–400 mg, *200–400* *mg*)		*P* = 0.43
Median VA of worst seeing eye logMAR (range, *IQR*)	0.22 (−0.10 to 1.80, *0.06 to 0.50*)	0.10 (−0.10 to 0.68, *0.00 to 0.17*)		*P* = 0.007
Median 10-2 field deficit of worst seeing eye (dB) (range, *IQR*)	−8.70 dB (−29.96 dB to −3.15 dB, −*18.78* *dB to −6.20* *dB*)	−3.73 dB (−27.04 dB to −1.81 dB, *−9.36* *dB to −2.29* *dB*)		*P* = 0.017
Disease distribution				
Parafoveal	73%	87%		
Pericentral	9%	11%		
Mixed	18%	3%		
ELM disruption on OCT imaging	67%	53%		*P* = 0.18
**Comparison of patients based on ELM status on OCT at diagnosis**				
	**ELM disruption at diagnosis**	**ELM preserved at diagnosis**		
Number	45	26		
Mean age in years (SD, *range*)	61.5 years (16.2 years, *28–94 years*)	59.9 years (13.5 years, *30–88 years*)		*P* = 0.67
Median VA of worst seeing eye, logMAR (range, *IQR*)	0.17 (−0.10 to 1.80, *0.08 to 0.32*)	0.10 (−0.10–0.50, *0.00 to 0.17*)		*P* = 0.03
Median field deficit of worst seeing eye (dB) (range, *IQR*)	−8.95 dB (−29.96 dB to −0.82 dB, *−18.15* *dB to −3.92* *dB*)	−3.67 dB (−20.67 dB to −0.05 dB, *−6.96* *dB to −2.75* *dB*)		*P* = 0.02
Symptoms at diagnosis	44%	31%		*P* = 0.33
Median duration of drug use in years (range, *IQR)*	14 years, 6 months (5 years to 50 years, *10 years to 20 years*)	10 years, 6 months (4 months to 40 years, *8 years to 16 years 3 months*)		*P* = 0.16
Median daily dose, mg (range, *IQR*)	400 mg (200–400 mg, *200–400* *mg*)	200 mg (66–400 mg, *200–400* *mg*)		*P* = 0.44

*SD* standard deviation, *IQR* interquartile range, *dB* decibels, *MD* mean deviation.

### Hydroxychloroquine dosing characteristics

The median daily hydroxychloroquine dose was 350 mg (non-parametric; inter-quartile range (IQR): 200–400 mg). Although most patients were taking 200 mg or 400 mg per day, some were on variable dosing regimens, in which case, the mean daily dose over the relevant intervals was calculated and recorded. The median duration of hydroxychloroquine therapy at diagnosis was 12.1 years (non-parametric; IQR: 9.1–20 years).

### Clinical characteristics

There was no significant difference in visual acuities (*p* = *0.29*, Wilcoxon matched-pairs signed rank test for non-parametric data) or 10-2 visual field sensitivities *(p* = *0.71;* Wilcoxon matched-pairs signed rank test for non-parametric data) between the right and left eyes (Table 2). Subsequent analyses used visual acuity and visual field data from the worse-seeing eye for each patient. Only 10-2 visual field testing data were included for analysis since it was the most frequently used test protocol, and parafoveal disease was the most common disease phenotype.

88% of patients (64/73) had at least 2 abnormal tests consistent with hydroxychloroquine retinopathy. 53% of patients had at least 3 abnormal tests. 87.2% of patients had abnormal SD-OCT imaging, 73% with abnormal fundus autofluorescence imaging, 94.5% (51/54) with abnormal visual field tests, 50% with fundoscopic signs (32/64). 17 patients had a full-field ERG of which 13 were abnormal and 13 had a multifocal ERG, all of which were reported as abnormal. 63% of patients had ELM disruption at diagnosis. The ratio of paracentral: pericentral disease: mixed disease was 50:6:7 (Table 2).

### Symptomatic versus asymptomatic patients

**Fig. 2:**
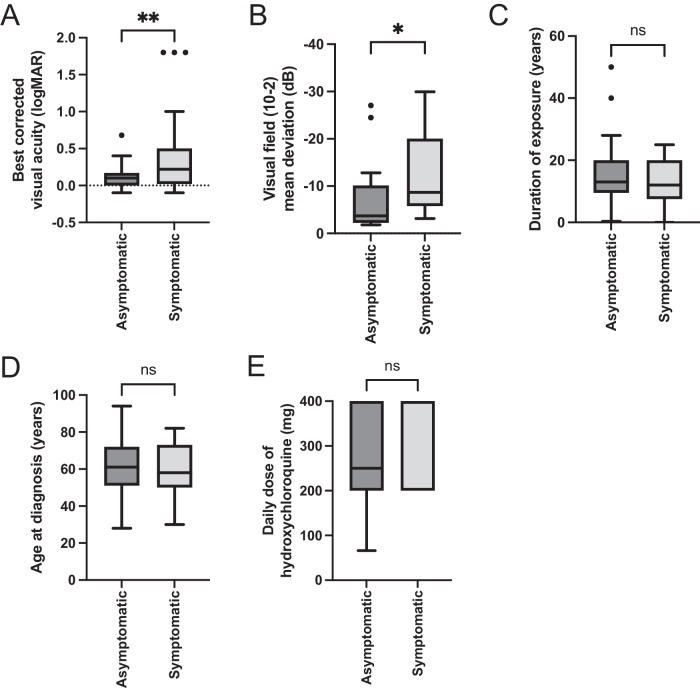
Comparison of visual function in symptomatic and asymptomatic patients with hydroxychloroquine retinopathy at diagnosis. **A** Visual acuity was significantly better at diagnosis in the asymptomatic patients detected as a result of monitoring, compared to those who presented with visual symptoms (median logMAR 0.1 vs. 0.22; *p* = 0.007; Mann–Whitney two-tailed test; *n* = *70*). **B** Mean deviation on 10-2 visual field testing was significantly preserved in the asymptomatic vs. symptomatic groups (*p* = 0.017; median MD −3.73 dB vs. −8.69 dB; Mann–Whitney two-tailed test; *n* = *34*). **C**–**E** No differences were observed between asymptomatic and symptomatic patients based on duration of hydroxychloroquine exposure (*p* = 0.33), age at diagnosis (*p* = 0.73) or daily dose (*p* = 0.43).

The ratio of asymptomatic to symptomatic patients at diagnosis was 43:27 (data missing for 3 patients). Asymptomatic patients had a significantly better visual acuity (median visual acuity symptomatic vs asymptomatic group: 0.22 vs 0.10, *p* = 0.006; Mann-Whitney unpaired t-test, non-parametric) and visual field sensitivity (median HVF deficit symptomatic vs asymptomatic group: −8.70 dB vs −3.73 dB, *p* = 0.01, Mann–Whitney unpaired t-test, non-parametric) compared to symptomatic patients at diagnosis (Fig. 2). Symptomatic and asymptomatic patients were otherwise matched in age at diagnosis (*p* = *0.72;* Welch’s unpaired t-test, parametric), duration of hydroxychloroquine use (*p* = *0.47*; Mann–Whitney unpaired t-test, non-parametric), hydroxychloroquine dose (*p* = *0.43*), and ELM disruption (*p* = *0.31;* Fisher’s exact contingency test for dichotomous variables of symptom and ELM disruption status).

Of the symptomatic patients, 63% complained of worse visual acuity *(n* = *17)*, 30% of scotoma *(n* = *8*; 4 patients reported both reduced visual acuity and a scotoma*)*, 7.4% of a deficit in colour vision *(n* = *2)*, and 18.5% had a variety of ‘other’ symptoms including glare (*n* = *1*), dry eye (*n* = *1*), photopsias (*n* = *2*) and floaters (*n* = *1*). The individual with dry eye also had other more relevant symptoms to toxic retinopathy.

### External limiting membrane (ELM) status on optical coherence tomography imaging

**Fig. 3:**
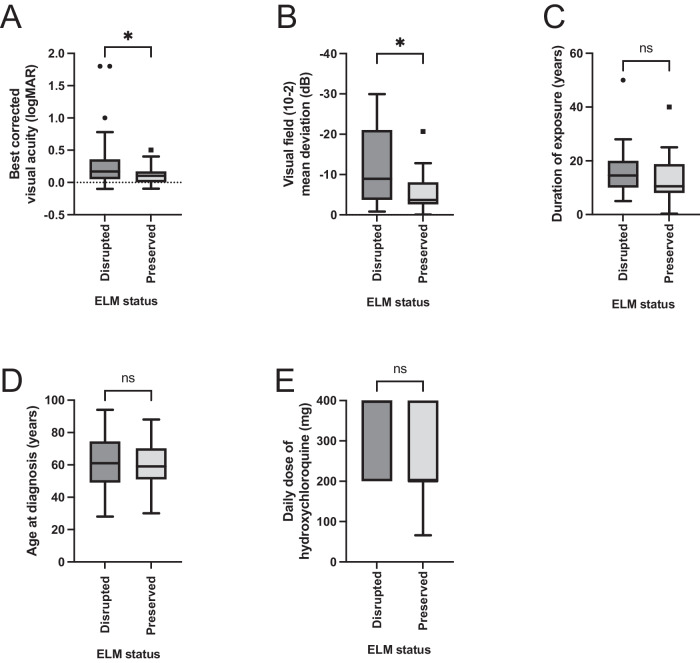
Comparison of visual acuity and visual field parameters based on the status of the external limiting membrane on OCT imaging at diagnosis. **A** Visual acuity was significantly preserved in those with ELM preservation vs. disruption (*p* = 0.027; median logMAR acuity 0.1 vs. 0.17; *n* = 71), as was visual function on 10-2 automated visual field testing (**B**; *p* = 0.024; median MD −3.67 dB vs. −8.95 dB; *n* = 41; both Mann–Whitney two-tailed test). There was no significant difference between groups in terms of duration of hydroxychloroquine exposure (*p* = 0.16), age at diagnosis (*p* = 0.67) and daily dose (*p* = 0.44).

Subgroup analysis identified that patients with ELM preservation on OCT imaging at diagnosis had significantly better visual acuity than patients with ELM disruption (median logMAR 0.1 vs 0.17, *p* = *0.03;* Mann–Whitney unpaired t-test, non-parametric) and a better preserved central visual field (median field sensitivity −3.67 dB vs −8.95 dB, *p* = 0.02, Mann–Whitney unpaired t-test, non-parametric). There was no difference in age at diagnosis (*p* = *0.67;* t-test with Welch’s correction, parametric), duration of hydroxychloroquine use (*p* = *0.16*, Mann–Whitney unpaired t-test, non-parametric), daily hydroxychloroquine dose (*p* = *0.44*, Mann–Whitney unpaired t-test, non-parametric), or presence or absence of symptoms *(p* = *0.17*, Mann–Whitney unpaired t-test, non-parametric*)* between patients based on ELM status on OCT imaging (Fig. 3; Table 2).

### Management

83% *(n* = *20)* were managed with cessation of hydroxychloroquine at diagnosis. 13% *(n* = *3)* were continued at the same dose pending rheumatological review (data unavailable for 1 case). There were no cases reported where a reduction in the daily dose of hydroxychloroquine was recommended.

### Follow-up

29 completed follow-up questionnaires were received (8 males, 21 females) with 11 asymptomatic and 14 symptomatic at the point of diagnosis (Supplementary Fig. 2). The mean duration of follow-up from diagnosis was 19.7 months (range: 3.7–51.7 months: standard deviation 13.4 months). There were no significant changes in visual acuity (OD: *p* = 0.18, OS: *p* = 0.32, Wilcoxon’s test, non-parametric, matched-pairs) or visual field mean sensitivity (OD: *p* = 0.13, OS: *p* = 0.81, Wilcoxon’s test, non-parametric, matched-pairs) between patients at diagnosis and at follow-up. One patient was eligible for registration as severely sight impaired, 3 as sight impaired (of which 1 had also had dry AMD), with 18 patients ineligible for registration as sight impaired at follow-up. Four patients were reported deceased, 2 lost to follow-up and 1 transferred to another unit.

## Discussion

Screening, or monitoring, for hydroxychloroquine retinopathy was recommended by the AAO (2011 & 2016) [17, 18] and the RCOphth (2018 & 2020) [19, 20]. At the time of the initial recommendation in the U.K., the number of long-term hydroxychloroquine users, and the number of individuals with definite retinopathy (RCOphth definition) [17, 20] in the general population were unknown. The number of long-term users of hydroxychloroquine in the U.K. has subsequently been approximated at 71,144 to 77,170 using two different methodologies [22]. Furthermore, recent data from three large real-world audits of monitoring outcomes in the U.K. suggest an aggregated frequency of retinopathy of 1.47% amongst long-term users using a standardised definition. These data predict approximately 1046 to 1135 long-term hydroxychloroquine users with definite retinopathy in the U.K. (diagnosed or undiagnosed).

Data from this study provide two further important estimates that are relevant to clinical commissioners at national and regional levels, and other key stakeholders. Firstly, we estimate the annualised frequency of detection of retinopathy in long-term hydroxychloroquine users was 0.04–0.08% during the study interval (1 in every 1247 to 2625 long-term users). Secondly, using the above estimates of individuals with retinopathy in the population, monitoring coverage was approximately 2.6–5.5% amongst long-term users during the initial study period (July 2018 to Feb 2020). This indicates that relatively few hospital eye services had established a monitoring service during this period; approximately 1999 to 3880 long-term hydroxychloroquine users were screened annually during this period. This study provides a mechanism to evaluate the national yield of retinal monitoring procedures in this at-risk group, and may be repeated in the future when monitoring is offered more broadly. It should be noted that a small minority of patients developed retinopathy with less than 5 years of drug exposure, supporting the earlier monitoring of those with additional risk factors.

Monitoring programmes for hydroxychloroquine retinopathy were recommended by the AAO and RCOphth on the assumption that monitoring procedures could detect pre-symptomatic retinopathy and preserve visual function at the point of disease detection. Here we show for the first time that visual function – as measured by visual acuity and central visual field testing - is better preserved at the point of diagnosis in a monitored, asymptomatic cohort compared to those who present with visual symptoms. The groups were otherwise matched for other key risk factors for retinopathy. This demonstration of efficacy is particularly important as the preservation of visual function is the primary benefit of monitoring for hydroxychloroquine retinopathy; with early diagnosis shown to reduce the likelihood of disease progression [6, 9–11]. Symptomatic and asymptomatic groups are likely to diverge in visual function over time beyond the point of diagnosis.

In the context of the data presented in this study - frequency of retinopathy, detection rates amongst long-term users, and detection rates of those with retinopathy in the population – the number of individuals that need to be screened in the at-risk population to detect one case is now better estimated. A cost-utility study may now be undertaken in the U.K. which may be a key determinant of the sustainability of monitoring; cost-utility data have been published in a U.S. context [23].

A raw population incidence of 0.44–0.86 per million per year in the total population and 0.56–1.09 per million per year in the adult population provides useful disease estimates for epidemiological comparison (i.e. similar to optic disc pit maculopathy and schisis retinal detachment [24, 25]). However, an increase in the coverage of monitoring may have a significant impact on the measured incidence of retinopathy - up to a maximum of 15.7–17.1 per million per year with universal monitoring coverage, based on current estimates of long-term users and available mainstream diagnostic tests recommended by the RCOphth monitoring guideline. Moreover, the development and application of more sensitive methods of disease detection and prediction in monitoring protocols may increase the diagnostic yield [26].

A large proportion of patients captured in this study had visual symptoms at the point of diagnosis: reduced visual acuity, scotoma, and altered colour vision. The high proportion of symptomatic patients (~39%) in this study reflect the low overall population coverage of monitoring at the time of the study. Colour vision disturbance is recognised as a recurrent feature of hydroxychloroquine retinopathy [27, 28], which is supported by findings of reduced central macular sensitivity on microperimetry [29]. Imaging studies have shown reduced macular pigment density in hydroxychloroquine retinopathy [30], which itself is associated with altered red-green colour discrimination [31]. The other reported symptoms likely represent reporting bias, rather than direct relevance to the retinopathy (i.e. volunteered on direct questioning). Moreover, since monitoring services were being established, there may have been a selection bias that favoured the referral of patients at the highest risk of retinopathy.

The integrity of the ELM on OCT imaging has been reported as a variable that has prognostic significance in hydroxychloroquine retinopathy [6, 11]. The data in this study show that ELM status relates to visual function with significantly reduced visual acuity and visual field sensitivity in patients with ELM disruption at diagnosis. It is unclear whether ELM disruption is a non-specific degenerative structural feature which correlates with poorer retinal function regardless of aetiology. ELM disruption was associated with photoreceptor cell loss on OCT imaging at follow up in one study of patients with hydroxychloroquine retinopathy, although this was not compared with measures of visual function [11]. The data shown in this study supports possible functional significance of ELM disruption on OCT imaging.

This study has some inherent limitations. Firstly, the diagnosis of hydroxychloroquine retinopathy could not be directly verified by the study investigators. However, the cohort generally had severe retinopathy with 50% of patients exhibiting signs on fundoscopy and 53% of patients with 3 abnormal tests, reducing the likelihood of clinical misclassification. A minority of patients (12%) had one abnormal test consistent with retinopathy which deviates from the definition of “definite retinopathy” as per RCOphth guidelines, and may introduce a small degree of error into the calculations. However, some variation in practice is typical of real-world studies. Secondly, calculations presented are predicated on the accuracy of published estimates of long-term hydroxychloroquine users, the frequency of retinopathy based on aggregated data from three relevant, published real-world studies, and the consequent number of estimated individuals affected by retinopathy. These data are the most accurate currently available and a range of error has been provided for all estimates. Adjustments were made for under-ascertainment and under-reporting to reduce the impact of these events .

## Summary

### What was known before:

- Hydroxychloroquine retinopathy is an increasingly recognised cause of bilateral, irreversible visual loss.
- The visual benefits of monitoring to detect early retinopathy remain unproven.
- The incidence, clinical characteristics, monitoring coverage and management of hydroxychloroquine retinopathy in the United Kingdom are incompletely characterised.

### What this study adds:

- The central visual field and visual acuity are better preserved at the point of diagnosis when retinopathy is detected during monitoring when compared to patients who present with visual symptoms.
- The approximate coverage of retinal monitoring in long-term hydroxychloroquine users in the U.K. was 2.5-5.5% during the study period.
- Hydroxychloroquine retinopathy was relatively severe at presentation within the cohort; drug cessation was recommended in every case.

## Supplementary information

Supplementary Figure 1. Temporal trends in case reporting.

Supplementary Figure 2. Flow diagram of patients included in the study.

Supplementary Table 1. Study questionnaires used for data collection

SUPPLEMENTARY MATERIALS
