# Supplementary figures and images for: The incidence, monitoring coverage and clinical characteristics of hydroxychloroquine retinopathy in the United Kingdom
Source: Eye (Lond). 2024 Jul 31;38(14):2796–804. doi: 10.1038/s41433-024-03168-0 (PMC11427468; doi:10.1038/s41433-024-03168-0)

**A**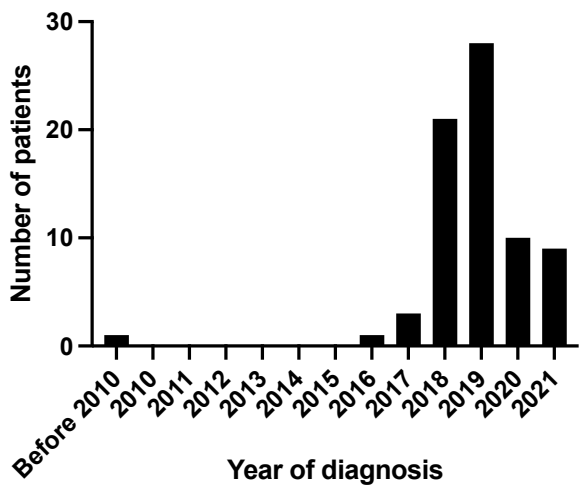**B**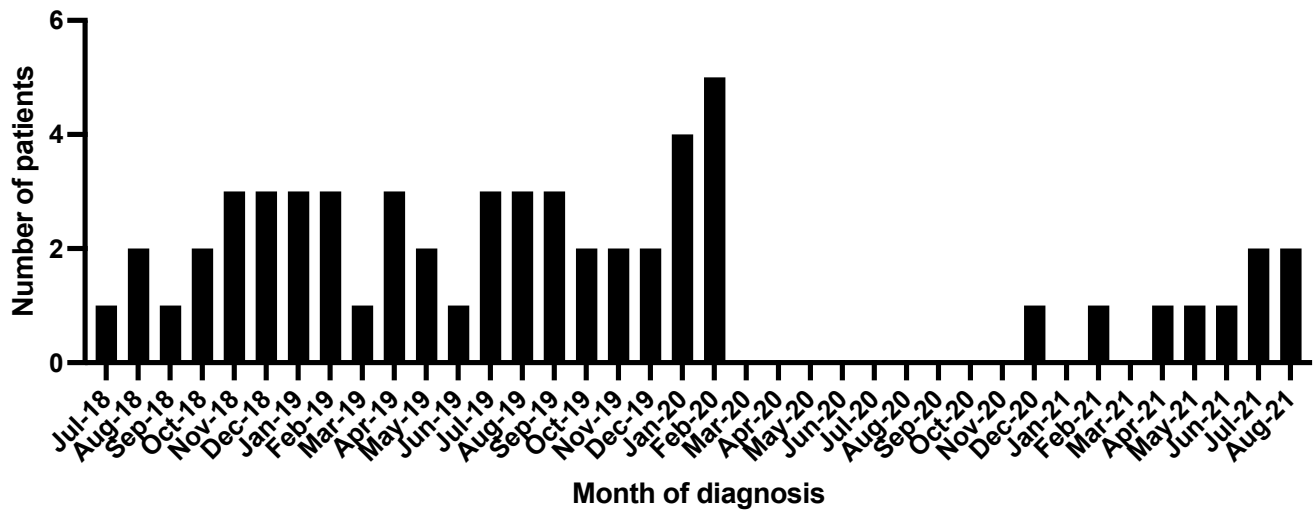

Supplement: Supplementary file 1 — Supplementary Figure 1. Temporal trends in case reporting. [file 41433_2024_3168_MOESM1_ESM.pdf]

**Supplementary Figure 2.** **Flow diagram of patients included in the study.**


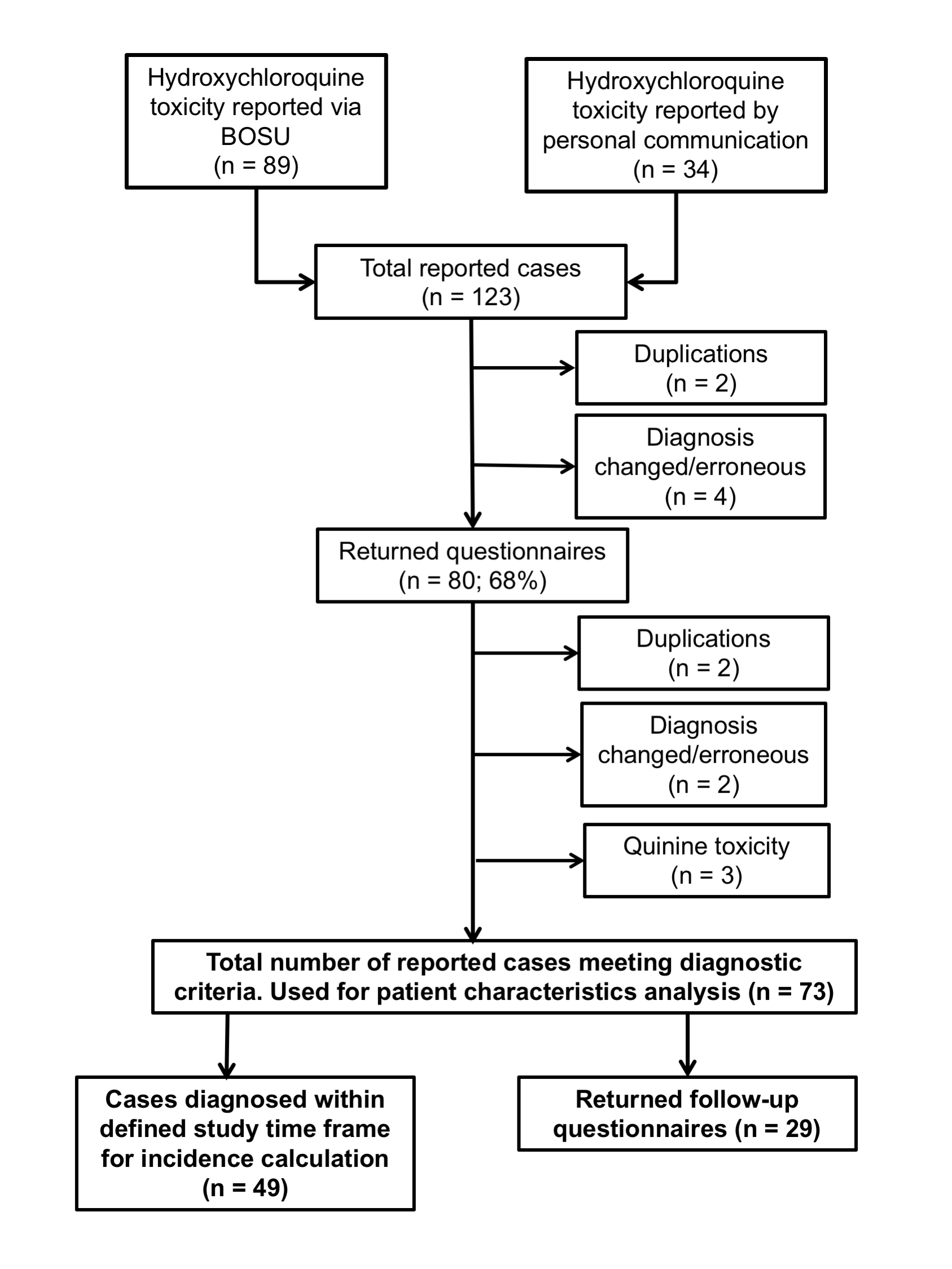

Supplement: Supplementary file 2 — Supplementary Figure 2. Flow diagram of patients included in the study. [file 41433_2024_3168_MOESM2_ESM.docx]
