# Supplementary material for: The incidence, monitoring coverage and clinical characteristics of hydroxychloroquine retinopathy in the United Kingdom
Source: Eye (Lond). 2024 Jul 31;38(14):2796–804. doi: 10.1038/s41433-024-03168-0 (PMC11427468; doi:10.1038/s41433-024-03168-0)
Supplement: Supplementary file 3 — Supplementary Table 1. Study questionnaires used for data collection [file 41433_2024_3168_MOESM3_ESM.pdf]

Supplementary Table 1. Study questionnaires used for data collection

1. Case details

Patient local hospital number: ______________

Month and year of birth: __________________

Patient Sex: ❑ Male ❑ Female

White	Asian or Asian British	Black or Black British	Chinese	Mixed Race	Other ethnic group
❑ British<br>❑ Irish<br>❑ Other (please specify)	❑ Indian<br>❑ Pakistani<br>❑ Bangladeshi<br>❑ Other (please specify)	❑ Caribbean<br>❑ African<br>❑ Other (please specify)	❑ Chinese<br>❑ Other (please specify)	❑ (please specify)<br>______________<br>__________	❑ Other (please specify)

Here are some questions about the diagnosis of hydroxychloroquine toxicity in this patient

2. What was the date of confirmation of the diagnosis of hydroxychloroquine retinopathy?

(Short case definition: macular dysfunction due to hydroxychloroquine toxicity confirmed on one investigation out of: automated visual field, OCT, autofluorescence or electrodiagnostics). For full case definition see Page 3 of this document.

________/________/________ *[DD/MM/YYYY]*

3. Does the patient have any relevant ocular co-morbidities?

☐ Cataract
☐ Diabetic maculopathy
☐ Glaucoma
☐ Age related macular degeneration
☐ Choroidal neovascularisation
☐ Macular oedema associated with vein occlusion
☐ Other, please specify:

1.__________________________ 2.__________________________ 3.__________________________

☐ Don't know

Next, we have some questions about indications and symptoms:

4. What is the patient's primary treatment indication for hydroxychloroquine?

☐ Systemic Lupus Erythematosus
☐ Rheumatoid Arthritis
☐ Other disorder (including dermatological). Please specify: ____________________________
☐ Don't know

5. At diagnosis, what symptoms did the patient have?

☐ Scotoma
☐ Reduced visual acuity
☐ Deficit in colour vision
☐ Other: please specify ________________________________
☐ Don't know
☐ Patient was asymptomatic

Next are some questions about the abnormalities on investigation of the patient

6. Vision at diagnosis

	RIGHT EYE	LEFT EYE
**Best corrected visual acuity** (Snellen or LogMAR)		
**Visual field Mean Deviation** (in decibels (dB))		

Please circle visual field protocol:<br><br>10-2 / 24-2 / 30-2 / not done		

7. What investigations were used to diagnose hydroxychloroquine retinopathy in this patient? Please tick a column for each investigation result in this case.

Investigation	Normal	Abnormal	Not needed	Not available
Fundoscopy				
Humphrey visual field				
Fundus autofluorescence				
Spectral domain OCT				
Electrodiagnostic testing:<br>ERG				
Electrodiagnostic testing:<br>Multifocal ERG				

8. Was the external limiting membrane disrupted on SD-OCT imaging at diagnosis?

☐ Yes
☐ No

9. What distribution of hydroxychloroquine retinopathy existed in this patient?
Please select A, B or C

A	B	C

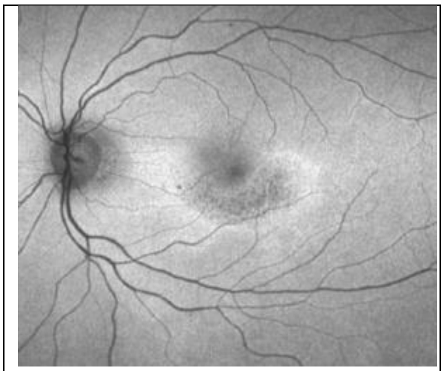	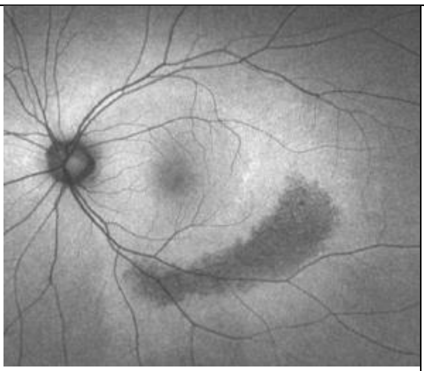	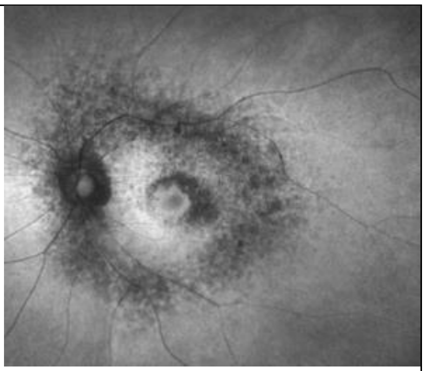
**Paracentral disease**	**Pericentral disease**	**Mixed: paracentral and pericentral disease**
retinal changes within a disc diameter of the foveal centre	retinal changes > 1 disc diameter away from the foveal centre	Paracentral AND pericentral distribution of disease

10. How long had this patient been taking hydroxychloroquine for?

_________ years ________months

☐ Don't know

11. What was the daily dose of hydroxychloroquine when the diagnosis was made?

_________ milligrams per day

☐ Don't know

12. Did this patient have known renal impairment?

☐ Yes
☐ No
☐ Don't know

13. Did this patient take tamoxifen in the past or at the time of diagnosis?

☐ Tamoxifen taken previously
☐ Tamoxifen taken at the time of diagnosis of hydroxychloroquine retinopathy
If known, what was the duration of tamoxifen use: _________years
☐ No history of tamoxifen use
☐ Don't know

14. How was this patient with hydroxychloroquine retinopathy managed at diagnosis?

☐ Hydroxychloroquine treatment was stopped
☐ Hydroxychloroquine dose was reduced: the new dose was _______mg per day
☐ Hydroxychloroquine was continued at the same dose

☐ Don't know

15. If hydroxychloroquine was continued, please specify the reason for this:

____

____

____

☐ Don't know

16. Was this patient referred to your ophthalmology department from another ophthalmology department?

☐ Yes. Please specify hospital referred from:______________________________
☐ No
☐ Don't know

Thank you for taking the time to complete this questionnaire.

Please return questionnaire to: Mr Imran Yusuf, Specialist Registrar in Ophthalmology, Oxford Eye Hospital, West Wing, John Radcliffe Hospital, Oxford, OX3 9DU.

Incomplete questionnaires should also be returned if outstanding clinical details cannot be identified.
