# Supplementary material for: The incidence, monitoring coverage and clinical characteristics of hydroxychloroquine retinopathy in the United Kingdom
Source: Eye (Lond). 2024 Jul 31;38(14):2796–804. doi: 10.1038/s41433-024-03168-0 (PMC11427468; doi:10.1038/s41433-024-03168-0)
Supplement: Supplementary file 4 — SUPPLEMENTARY MATERIALS [file 41433_2024_3168_MOESM4_ESM.docx]

**SUPPLEMENTARY MATERIALS**

**Supplementary Figure 1. Temporal trends in case reporting.**

Reporting of hydroxychloroquine retinopathy cases by **(A)** year and **(B)** month of diagnosis.

**Supplementary Figure 2.** **Flow diagram of patients included in the study.**

**Supplementary Table 1.** **Study questionnaires used for data collection**
